# Years of life lost by CNCD attributed to occupational hazards in Brazil: GBD 2016 study

**DOI:** 10.11606/S1518-8787.2020054001257

**Published:** 2020-03-12

**Authors:** Ada Ávila Assunção, Elisabeth Barboza França

**Affiliations:** I Universidade Federal de Minas Gerais Faculdade de Medicina Programa de Pós-graduação em Saúde Pública Belo HorizonteMG Brasil Universidade Federal de Minas Gerais . Faculdade de Medicina . Programa de Pós-graduação em Saúde Pública . Belo Horizonte - MG , Brasil

**Keywords:** DALY, Occupational hazard factors, Brazil, Workers’ health

## Abstract

**OBJECTIVE:**

To assess the years of life lost due to premature death and *disability-adjusted life years* (DALY) as a result of chronic noncommunicable diseases attributable to occupational hazard factors, and to compare their position according to the risk ranking for chronic noncommunicable diseases in 1990 and 2016.

**METHODS:**

Data for the DALY indicator, estimated from the Global Burden of Disease 2016 (GBD 2016) study, were analyzed for noncommunicable chronic diseases attributable to occupational, and other risk factors, selected in Brazil. A descriptive analysis was performed comparing the proportion of DALY by sex and age group (15 to 49 and 50 to 69 years old), as well as the ranking of occupational hazard factors in 1990 and 2016.

**RESULTS:**

In 2016, ergonomic risk factors, carcinogenic agents, and noise in the workplace were among the 25 largest contributors to DALY for chronic noncommunicable diseases affecting the age group between 15 and 49 years. The contribution of all occupational hazard factors increased in 2016, except for occupational aerodispersoids affecting men. Concerning the age group between 50 and 69, occupational carcinogens stand out, with an increase of 26.0% for men, and 17.1% for women in 2016. Risk factors evaluated according to their 1990 and 2016 ranking show that occupational hazards have all scored higher on the second evaluation (2016), especially when compared with other risks.

**CONCLUSIONS:**

The global burden of chronic noncommunicable diseases attributed to occupational hazard factors has become increasingly important. We suggest the strengthening of the approach of occupational hazard factors in the agendas for tackling these diseases in Brazil.

## INTRODUCTION

Noncommunicable chronic diseases (NCD) cause 41 million deaths worldwide. Each year, 15 million people aged 30 to 69 die from cardiovascular disease, cancer, chronic respiratory disease, and diabetes. Poor/inadequate diet, physical inactivity, alcohol abuse, and smoking are behaviors that increase the risk of CNCD. These modifiable risk factors are addressed in Brazil by the Strategic Action Plan to Combat CNCD ^a^ , which is in line with the guidelines of the World Health Organization (WHO) ^1^ .

CNCD are disabling. Under the Brazilian Social Security (BSS), in 2016, musculoskeletal diseases were the main cause of leave from work, which justified 407,000 sick leaves, followed by digestive tract diseases (279,000), mental illness (185,000), neoplasms (165,000), and diseases of the circulatory system (159,000) ^b^ .

There is evidence of an association between these diseases and occupational hazard factors ^2 , 3 , 4 , 5^ . In general, they are cross-sectional studies conducted on samples of workers from a branch or production sector (the plastics industry ^2^ , for example) or specific occupational categories such as bus drivers ^3^ or healthcare professionals ^5^ . These and other studies were commented in a recent review article, in which the authors systematized results of occupational surveys conducted in the last decade to obtain an overview of both the contribution and the gaps in knowledge about the health of Brazilian workers, as well as indicating challenges in the public health field ^6^ . Among the gaps and challenges are the difficulties in estimating risks due to multiple exposures in the workplace, as well as the under-registration related to the current labor market configuration. The complexity related to multiple exposures, in which combination and sometimes synergy of effects occurs, requires sophisticated statistical designs and methods when the objective is to identify risks and monitor work situations. For these and other reasons, the burden resulting from occupational hazards is not yet sufficiently measured or broadly characterized ^7^ . As for under-registration, its origin lies within the incompleteness or lack of information on health profiling, employment characteristics, and occupational exposure. The coverage gaps in the employment information system are marked by the increasing proportion of people employed without a formal contract: 82.9% to 50.7%, as observed in Maranhão and Santa Catarina, respectively ^c^ . Knowing that 24.2% of Brazilian adults have more than one chronic disease ^8^ , how does one obtain quality information to formulate actions to monitor and prevent CNCD associated with occupational hazard factors?

The study Global Burden of Disease (GBD) has produced comprehensive assessments of human health since the 1990s. The main metric used in the GBD study was the disability-adjusted life years (DALY). This indicator, in a single measure, expresses the years lost due to illness, that is, the years of life lost due to premature death, and the unhealthy years spent living with a disability. The calculation of this indicator is based on a scale ranging from zero to one, where zero designates the state of full health and one designates death, the worst possible state of health. Life years lost due to death are calculated by reference to life expectancy estimated from the lowest mortality rates for each age group in locations with more than five million inhabitants. The years of life lost (YLL) are added to the years lived with disability (YLD) employing a scale that associates mortality, disease, and their sequelae ^9^ .

In addition to measuring health loss, the GBD study examines three risk factor (RF) groups. For each of those selected, the population attributable risk (PAR) is calculated, which measures the disease burden attributable to certain exposures ^10^ . PAR allows the identification, for example, of what index of the total risk for fatal lung cancer in the general population is due to occupational hazards.

This study aimed at using estimates from the GBD 2016 study to assess healthy years of life lost due to premature death and CNCD disability attributable to occupational hazard factors and comparing their position in risk ranking in 1990 and 2016.

## MATERIAL AND METHODS

### Research Context

The conceptual, methodological and operational advancement promoted by the GBD study results from the institutional efforts of 195 countries and territories, in addition to having powerful computational resources (big data) and funding provided by the Bill & Melinda Gates Foundation ^9^ . To identify both health risks and health degradation by disease and injury, the study elaborates on complex strategies for combining data from different sources. The selected risks are evaluated in an articulated manner, to identify them according to age, sex, and geographical area.

Since the Disease Burden workshop in Brazil in 2014, public health professionals and researchers have been involved in the partnership between the University of Washington Institute for Health Metrics and Evaluation (IHME) ^10^ , the Brazilian Ministry of Health, and the Universidade Federal de Minas Gerais (UFMG). The use of the metrics elaborated by the GBD study was one of the impulses for Brazilian institutions in joining this network ^11^ . The dissemination of the methodology reached the academic environment and health services to motivate, among others, the elaboration of this article.

### GBD Study Estimates

The GBD study estimates result from a complex process of modeling primary sources as detailed in previous publications ^12^ . Specific software was developed to estimate mortality (CODEM) and morbidity (Dis-mod), as well as technical devices for covariate adjustments (GDP, education, etc.). Estimates are shown with their respective uncertainty ranges. On the IHME ^10^ website, the user can access all these estimates calculated through standardized methodology. According to the researcher’s interest, it is possible to access data available from 195 countries, 20 age groups, both sexes, and three risk groups: environmental/occupational, metabolic and behavioral. The user has the option of choosing to view schematics and illustrative figures on the information according to year of observation, risk, and cause group.

As previously explained, the GBD study is concerned with population attributable risks (PAR), which calculate how the disease burden would have been reduced if exposure had been changed to a minimum level in the past, the so-called theoretical minimum risk exposure level (TMREL). TRMEL is considered an advanced construct as it uses the minimum exposure regarding the lowest theoretical exposure level. In addition to TMREL, PAR is based on two other components: relative risk and the prevalence of risk factors within the population. Relative risk estimates are based on consistent research results, such as randomized controlled trials, cohort studies, and others, providing they had been developed using appropriate methods ^9^ . Exposure levels and relative risks for each of the listed factors are measured according to available literature ^12^ . The prevalence of RF is estimated according to information from different types of surveys, such as those conducted at the respondent’s home, or environmental measurements collected by different strategies, including satellites. Chart 1 shows the GBD study definitions for each occupational RF.

**Chart 1:** Occupational hazard factor definitions assessed by the GBD study 13

Occupational asbestos	Accumulated occupational exposure to asbestos according to the mortality rate from pleural mesothelioma.
Bronchoconstrictor substances in the occupational environment	The proportion of individuals exposed to bronchoconstrictors in the occupational environment based on population distribution in nine economic sectors.
Occupational carcinogens	The proportion of individuals in groups identified as exposed (high and low exposures) to recognized carcinogenic agents (arsenic, acids, benzene, beryllium, cadmium, chromium, diesel, formaldehyde, nickel, polycyclic aromatic hydrocarbons, passive smoking, silica, trichloroethylene), using as reference the population distribution in 17 economic sectors.
Accidents at work	The proportion of fatal accidents attributed to work activity in seventeen economic sectors, regarding the rates recorded in each sector.
Ergonomic factors	The proportion of individuals exposed to low back pain risk factors, based on population distribution in nine economic sectors.
Occupational noise	The proportion of individuals exposed to a sound intensity level exceeding 85 decibels in the occupational environment, using as reference the population distribution in 17 economic sectors.
Aerodispersoid particles in the occupational environment	The proportion of individuals to a aerodisersoid particles, based on population distribution in 17 economic sectors.

### Analysis Presented by this Article

This descriptive study used the global burden of disease estimates for Brazil found on the GBD 2016 study, whose data are publicly available from the IHME ^10^ website, where all indicators are calculated and updated. The focus of this study was to observe the DALY indicator for CNCD, according to RF categorized up to the third level ^12^ . The proportions of healthy years lost for men and women were examined separately for two age groups: 15 to 49 years, and 50 to 69 years. This strategy enabled us to obtain the ranking of RF, which contributed most to the CNCD-related DALY indicator and the comparison of the 2016 results with those of 1990.

As CNCD, the GBD study includes cardiovascular diseases; malignant neoplasms; other malignancies; respiratory diseases; diabetes mellitus; musculoskeletal disorders; skin disorders; digestive disorders; mental and nervous system disorders; sensory organ disorders; genitourinary disorders; neurological conditions; congenital anomalies; oral conditions; and endocrine, blood and immunological dysfunctions ^13^ . It is noteworthy that data from the GBD 2016 study on morbidity for Brazil were extracted from national surveys, such as the National Health Survey (PNS), the Surveillance System for Risk and Protective Factors for Chronic Diseases by Telephone Survey (Vigitel), and the National Household Sample Survey (PNAD), among others, totaling 118 sources. For sources of occupational exposure, results from national workforce surveys, demographic studies, information from international systems on occupational exposure to carcinogens, data on occupational accidents available from the International Labor Organization database, and noise information were consulted, obtained from specific industrial surveys ^13^ .

The GBD Brasil project was approved by the Research Ethics Committee of the Universidade Federal de Minas Gerais, CAAE 62803316.7.0000.5149.

## RESULTS

**Figure 1:**
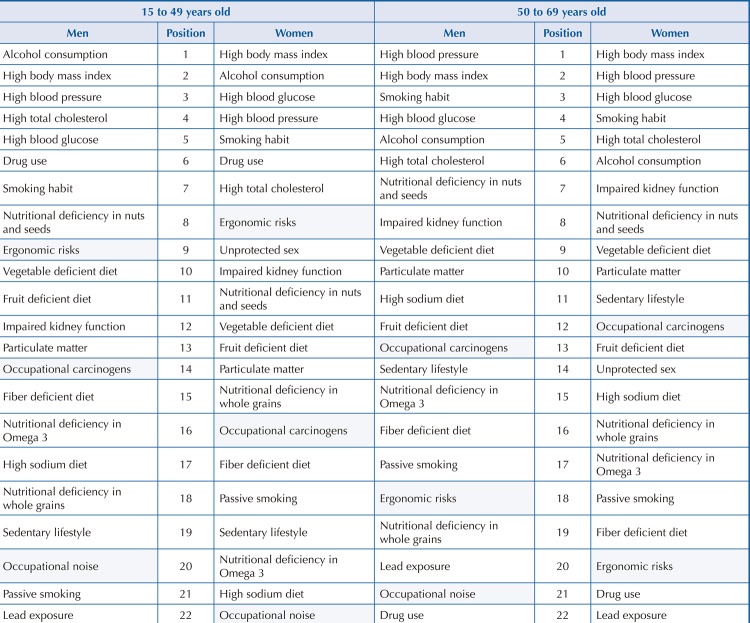
Major risk factors for noncommunicable diseases according to disability-adjusted life years (DALY) and to sex and age group. Brazil, 2016.

In 2016, within the age group 15 to 49 years considered productive, alcohol consumption and high BMI were the two main risk factors for DALY by CNCD. For men, alcohol consumption prevailed (13.28%), and for women, high BMI (7.77%). Concerning occupational hazards, ergonomic factors, carcinogenic agents, and noise stood out, in this order, for both sexes ( Figure 1 ).

Among men aged 15 to 49 years, ergonomic factors (2.57% of DALY) rose from 15th position in 1990 to 9th position in 2016, showing a 25.47% increase in DALY. It is noteworthy that only the increase related to sodium-rich diets (84.64%) and drug use (30.7%) was greater than that related to ergonomic RF ( Figure 2A ). Among women ( Figure 2B ), the RF (2.11%) rose from 14th to 8th position, corresponding to the largest increase (22.39%) after the high-sodium diet (40.99%). Occupational carcinogenic agents (1.23%) among men increased from 19th to 14th position, showing an increase of 11.89% compared to 1990 ( Figure 2A ). Among women, the increase in total DALY was 9.54%, causing the change from 20th to 16th position in 2016 ( Figure 2B ). In 2016, noise was the third occupational hazard in the ranking, which rose from 21st to 20th position among men ( Figure 2A ), and from 24th to 22nd among women ( Figure 2B ) in the age group between 15 and 49 years. Compared with 1990, the increase in total DALY by CNCD attributable to occupational noise was greater for women (20.38%) than for men (11.9%).

**Figure 2A:**
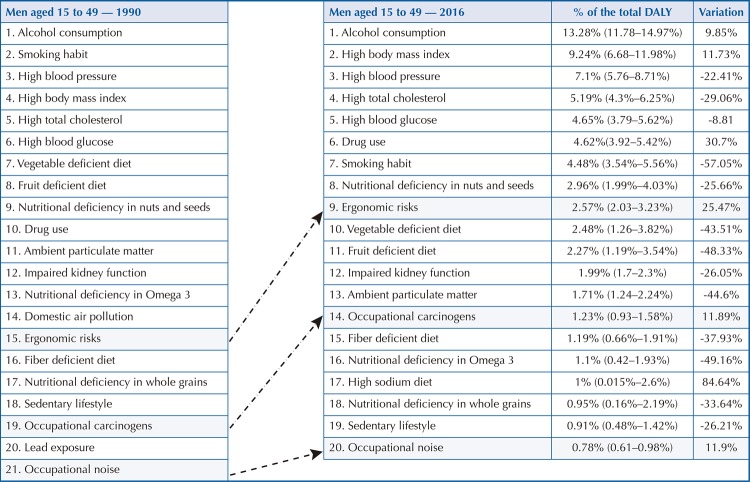
Risk factors that most contributed to premature death and loss of health from chronic noncommunicable diseases among men aged 15 to 49. Brazil, 1990 and 2016 DALY: disability-adjusted life years

**Figure 2B:**
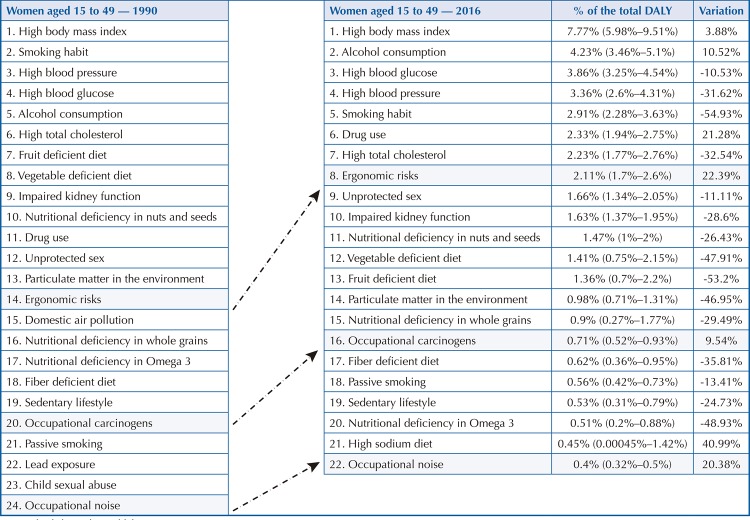
Risk factors that most contributed to premature death and loss of health due to chronic noncommunicable diseases among women aged 15 to 49. Brazil, 1990 and 2016 DALY: disability-adjusted life years

In the age group between 50 and 69 years, high blood pressure and BMI are the main risks for DALY by CNCD. For men, high blood pressure (19.55%) is the most relevant RF. For women, it is a high BMI (17.1%). In this range, the following occupational hazards stand out in this order for both sexes: occupational carcinogenic agents, ergonomic factors, and noise ( Figure 1 ).

Among men aged 50 to 69, occupational carcinogenic agents (2.97%) rose from 16th position in 1990 to 13th position in 2016 ( Figure 3A ), with a DALY increase of 26.04%. Among women in this age group, these risks (1.95%) rose from 19th to 12th position ( Figure 3B ), showing an increase of 17.1%. As for ergonomic factors, among men (1.18%), they rose from 22nd to 18th position ( Figure 3A ), an increase of 30.7%. Among women (1.06%), the change went from 22nd to 20th position ( Figure 3B ), an increase of 14.43%. The third occupational hazard is noise, which in 2016 rose from 23rd to 21st position among men, an increase of 19.27% if compared with 1990 ( Figure 3A ). Among women aged 50 to 69, the position changed from 25th (1990) to 23rd place (2016), an increase of 19.39% ( Figure 3B ).

**Figure 3A:**
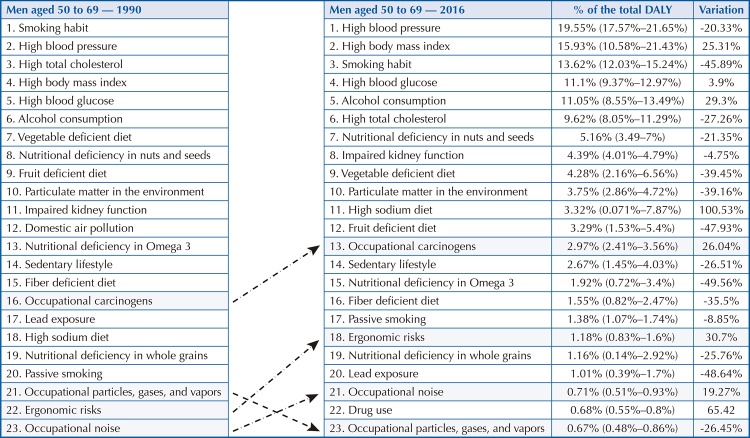
Risk factors that most contributed to premature death and loss of health from chronic noncommunicable diseases among men aged 50 to 69. Brazil, 1990 and 2016 DALY: disability-adjusted life years

**Figure 3B:**
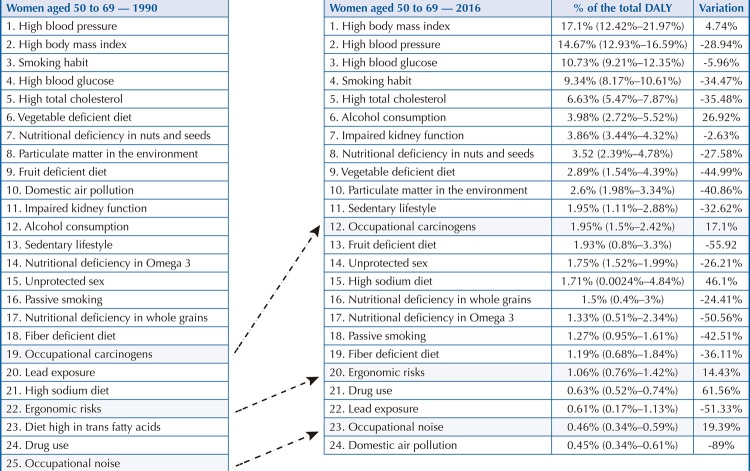
Risk factors that most contributed to premature death and loss of health from chronic noncommunicable diseases among women aged 50 to 69. Brazil, 1990 and 2016. DALY: disability-adjusted life years

Aerodispersoids (gases, vapors, and particles in the environment) configured relevant RF only to men aged 50 to 69 in 1990. However, there was a decrease in this RF from 21st to 23rd position.

In summary, in 1990 and 2016, the main occupational RF for CNCD was ergonomic for both men and women aged 15 to 49 years. When analyzing the age group between 50 and 69 in 1990 and 2016, the main occupational hazard factor for CNCD for both sexes are occupational carcinogenic agents.

## DISCUSSION

Using estimates from the GBD study, in 2016 there was an increase in the healthy years of life lost by CNCD attributable to occupational hazards in comparison with 1990. The relevance of ergonomic risks, carcinogenic agents, and noise was not surprising, considering the known association between these occupational hazards and illnesses ^2 , 3 , 4 , 5^ . There is also evidence of the correlation between these risks and disease burden in other countries ^14 , 15 , 16^ . The differences observed when examining the healthy years lost according to sex confirm hypotheses attributable to the sexual division of labor ^17^ .

In Iran, ergonomic factors and noise topped the list of occupational hazards that had most contributed to the total DALY in 2015 ^16^ . In Spain, occupational carcinogens ranked 12th in the ranking of environmental risks related to the total DALY in 2016 ^15^ . In India, occupational hazards ranked third when calculating healthy years lost due to chronic obstructive pulmonary disease in 2016 ^14^ . Disparities in comparisons can be attributed to a set of factors that vary from country to country — differences in the quality of national information systems, for example. In the Iranian study, estimates did not include exposure to carcinogenic trichloroethylene because this information was not available from any reliable source in the country ^16^ . Regarding the study carried out in Spain, Soriano et al. ^15^ presented a health situation summary according to the GBD study estimates for the country in 2016 for all 333 diseases and injuries, thus differing from our study, which focused only on CNCD. In the Indian study , estimates are exclusively related to chronic obstructive pulmonary disease (COPD) and asthma ^14^ . It is noteworthy that there should be necessary caution when interpreting disparities, once the specific limits that influence studies on respiratory diseases and other CNCD are known, such as the lack of consensus on case definition and the multiplicity of procedures involved in the application of spirometry, in addition to the bias arising from impediments in access to diagnostic services. At the technical level, complex procedures for the measurement of the exposed population are recommended when the disease presents a long latency period, as it happens with cancer. National capacities concerning such inputs also vary. Besides, errors in exposure assessment may influence the results of a national study ^18^ . This set of factors would explain the differences in estimates, at least in part. The limits of the GBD study will be presented ahead, which should be recalled when analyzing the contrast of inter-country results.

Macro-structural factors would explain the differences in the ranking of occupational hazards. National peculiarities regarding employment turnover and life expectancy are known, which influence the age at entry and exit to the workforce, with repercussions on the size and vulnerability of the population exposed to occupational carcinogens, for example. Despite disparities when comparing national results, consistency when examining correlated data (PS information on causes for work leaves, for example) is indispensable evidence for interpreting the results described. As mentioned, sickness allowances due to musculoskeletal causes, which are commonly associated with ergonomic factors in the adult population, predominated in 2016 ^19^ . Thus, there is a coherence between disease burden attributable to ergonomic risks and musculoskeletal diseases as the first cause of work leave recognized and declared by the PS in 2016, as indicated by the results of our study.

Sex differences explain the workforce composition according to sex, which is called the sexual division of labor ^17^ . In Brazil, 15.6% of employed men were working in positions related to civil construction in 2014. Industries absorbed 17.2% of employed men, and 10.6% of employed women. In the service sector (education, health, and social services), the proportion was reversed: 4.3% of employed men, compared with 18.2% of women in this situation. Regarding employed women, the majority performed domestic services (13.9%) or worked in the educational sector (7.4%). Thus, despite the increasing participation of women in the workforce, they remain a minority in the heavy metallurgical industry, mining, construction, and agriculture, to name a few ^d^ . This sexual division of labor reproduces male and female identity configurations. Men predominate in industries where tasks require more physical strength and contact with chemicals. Women are the majority in the service sector characterized by care-giving. In industry, they occupy positions in sectors characterized by the most thorough and repetitive operations, such as microelectronics ^17^ . This distribution of tasks and positions is expected to coincide with the distribution of exposure to occupational hazards specific to both sectors, which elucidates the differentials of the disease burden attributable to these risks according to sex ^20^ . Similarly, the ranking of occupational hazards was affected by sex in other countries as well ^14 , 15 , 16^ .

We observed a higher relevance in the ranking of risks regarding carcinogenic agents for men than for women, especially within the younger age group. Once again, the results are best interpreted based on the hypotheses of the sexual division of labor ^20^ because most carcinogens found in the workplace are chemicals (asbestos, benzene, and organophosphates, for example), mainly handled in heavy metallurgy, mining, construction, and the agricultural sector, mostly composed of male workers ^4^ . The relevance of carcinogenic risks over other occupational hazards was observed regardless of sex within the age group between 50 and 69. The slow process of carcinogenesis triggered by continued occupational exposure to such agents ^e^ could explain the progressive increase in incidence and mortality rates according to age for both men and women.

Noise is the third occupational RF identified. The results are consistent with the World Health Organization classification ^f^ , which ranks noise third among occupational hazards. Engines, pumps, ventilation systems, etc., are known for producing high intensity and low-frequency noise; mainly in the transport, industry, mining, construction, and agricultural sectors ^21^ . The effects of noise and extra-auditory function alterations induced by noise produced in facilities of the mentioned sectors, and others, are associated with the prevalence of CNCD among those exposed ^22^ .

Could the increase in the burden of illness attributable to occupational hazards for CNCD in 2016 be the effect of the period marked by institutional, social, and economic changes that modified working environments and employment bonds? At the institutional level, there has been a substantial transformation of social policies in Brazil in recent decades, leading to the advancement of knowledge and health care, including workers’ health. The increase in access and improvement in the quality of information systems are the result of these policies, which favor the diagnosis of events and the registration of hazards ^1^ . It is possible, however, that the increased contribution of occupational hazards for the disease burden is, in line with the hypotheses of the present research, a consequence of the deterioration of working environments with innovations in production processes and precarious labor relations. At the macrostructural level, globalized capitalism with increasing frequency, confronting workers and jobs.

Changes in working environments with the introduction of new technologies, substances, and work processes are as fast as commercial design and the incentive for the consumption of new products worldwide ^23^ . This transformation is accompanied by exposure to new risks and increased exposure to risks already known ^7^ .

Data available from the GBD study for Brazil enabled us to estimate the impact of occupational hazards on CNCD in Brazilian adults in an unprecedented way, using powerful and innovative metrics and constructs (DALY and PAR, for example), to circumvent some limits of classical studies ^6^ . By bringing together national data sources, especially household survey results, as well as satellite measurements, the GBD study created opportunities for researchers and public health professionals. However, it is important to inform the known limits of the GBD study. Risk estimates may be resulting in underestimation of the effect, and, therefore, of the load itself ^24^ . Firstly, it is known that national information on the number of exposed individuals and exposure levels is generally incomplete or nonexistent, either because of the heterogeneity of the workforce distribution or the diversity of arrangements in production sectors and subsectors in which exposure to multiple agents adds complexity to risk measurement ^4 , 7 , 25^ , as previously explained. Secondly, while monitoring for occupational hazard factors, environmental measurements, and periodic control of potentially exposed workers is provided for in the regulatory framework ^g^ , there are no information systems for occupational exposure in Brazil. Employment turnover and the effect of the healthy worker constitute, in the third place, another barrier, because there is a tendency to exclude those unhealthy to favor the demands of production. The turnover explains the exposure dispersion, given the fact that the worker will probably navigate between companies or sectors ^4 , 6 , 25^ . Also, it is internationally recognized that there is an insufficient intellectual, academic, and financial investment required to identify the occupational burden of disease. In this scenario of employment dynamics and information scarcity, the elaboration of workers’ health surveillance measures is impaired, with repercussions on the chances of health deterioration ^7^ .

The relevance of occupational hazards in the disease burden, as evidenced by the results presented, is in line with previous publications both in Brazil ^26^ and in other countries ^14 , 15 , 16^ . The 12 national targets of the Strategic Action Plan to Combat Noncommunicable Chronic Diseases (2011–2022), however, do not include an approach for occupational exposure. The National Worker Health Policy ^h^ , in turn, makes explicit the intention to align itself with the set of health policies within the Unified Health System, once work is admitted as one of the determinants of the health-disease process ^7 , 27^ . Unpublished results on the impact of CNCD attributable to occupational hazards are likely to raise debate about the desired occupational health actions crosscutting, and provide elements for shaping occupational-related axes in upcoming policy updates to monitor and prevent CNCD.

## CONCLUSION

When analyzing the three groups of risk factors for DALY by CNCD in 1990 and 2016, there was a rise in the position of occupational hazards for both sexes concerning the other RF assessed by the GBD study. The emphasis given to occupational RF is probably related to structural changes in the labor market and production processes. The sexual division of labor explained differences in RF ranking according to gender. The results presented are in line with the current knowledge regarding the association between CNCD and occupational exposure; also converge with the causes of disability affecting adults covered by social security in the same period. Therefore, there should be a development of occupation-related pathways in future policy updates in order to monitor and prevent CNCD.

## Footnotes

a Brasil. Ministério da Saúde. Secretaria de Vigilância em Saúde. Departamento de Análise de Situação de Saúde. Plano de ações estratégicas para o enfrentamento das doenças crônicas não transmissíveis (DCNT) no Brasil 2011-2022 / Ministério da Saúde. Secretaria de Vigilância em Saúde. Departamento de Análise de Situação de Saúde. – Brasília: Ministério da Saúde, 2011. Acesso 28 Ago 2018.

b Ministério da Fazenda. Anuário Estatístico da Previdência Social/Ministério da Fazenda. Secretaria de Previdência, Empresa de Tecnologia e Informações da Previdência. Brasília: MF/DATAPREV, 2016. Acesso 22 Fev 2019.

c Brazilian Institute of Geography and Statistics. Summary of social indicators: an analysis of the living conditions of the Brazilian population: 2016 / IBGE, Population Coordination and Social Indicators. – Rio de Janeiro: IBGE, 2016. Access Feb 24, 2019.

d Departamento Intersindical de Estatística e Estudos Socioeconômicos (DIEESE). Anuário do Sistema Público de Emprego, Trabalho e Renda 2015: mercado de trabalho. São Paulo: DIEESE, 2015.

e Brasil. Ministério da Saúde. Secretaria de Vigilância em Saúde. Departamento de Vigilância Ambiental e Saúde do Trabalhador. Atlas do câncer relacionado ao trabalho no Brasil. Brasília. Ministério da Saúde, 2019. Disponível em: http://bvsms.saude.gov.br/bvs/publicacoes/atlas_cancer_relacionado_trabalho_brasil.pdf . Acesso 24 Fev 2019.

f WHO - World Health Organization. Global Health Risks: Mortality and burden of disease attributable to selected major risks. WHO: Geneva, 2009. Acesso 12 Set 2018.

g Brasil. Ministério da Saúde. Secretaria de Vigilância em Saúde. Departamento de Vigilância Ambiental e Saúde do Trabalhador. Atlas do câncer relacionado ao trabalho no Brasil. Brasília. Ministério da Saúde, 2019. Disponível em: http://bvsms.saude.gov.br/bvs/publicacoes/atlas_cancer_relacionado_trabalho_brasil.pdf . Acesso 24 Fev 2019.

h Brasil. Ministério da Saúde. Portaria nº 1.823, de 23 de agosto de 2012: Institui a Política Nacional de Saúde do Trabalhador e da Trabalhadora. Disponível em: http://bvsms.saude.gov.br/bvs/saudelegis/gm/2012/prt1823_23_08_2012.html . Acesso em 24 Fev 2019.
