# Supplementary material for: Years of life lost by CNCD attributed to occupational hazards in Brazil: GBD 2016 study
Source: Rev Saude Publica. 2020 Mar 12;54:28. doi: 10.11606/S1518-8787.2020054001257 (PMC7069713; doi:10.11606/S1518-8787.2020054001257)
Supplement: Translation

# Translation: Anos de vida perdidos por DCNT atribuídos aos riscos ocupacionais no Brasil: estudo GBD 2016

**Authors:** Ada Ávila Assunção, Elisabeth Barboza França

**Affiliations:** I Brasil Universidade Federal de Minas Gerais. Faculdade de Medicina. Programa de Pós-graduação em Saúde Pública. Belo Horizonte-MG, Brasil

**Keywords:** DALY, Fatores de Risco Ocupacionais, Brasil, Saúde do Trabalhador

## Abstract

**OBJETIVO:**

Avaliar anos de vida perdidos por morte prematura e por incapacidade ( *disability-adjusted life years* – DALY) em decorrência de doenças crônicas não transmissíveis atribuíveis a fatores de risco ocupacionais e comparar a posição desses fatores no ranking dos riscos para doenças crônicas não transmissíveis em 1990 e 2016.

**MÉTODOS:**

Os dados referentes ao indicador DALY, estimado no estudo de Carga Global de Doenças de 2016 (GBD 2016), foram analisados para doenças crônicas não transmissíveis atribuíveis a fatores de risco ocupacionais e outros selecionados no Brasil. Realizou-se análise descritiva comparando a proporção de DALY por sexo e faixa etária (15 a 49 e 50 a 69 anos), além do ranqueamento de atribuição dos fatores de risco ocupacionais em 1990 e 2016.

**RESULTADOS:**

Em 2016, fatores de risco ergonômicos, agentes carcinogênicos e ruído no ambiente laboral estiveram entre os 25 que mais contribuíram para os DALY por doenças crônicas não transmissíveis na faixa etária de 15 a 49 anos. A contribuição de todos fatores de risco ocupacionais aumentou em 2016, exceto os aerodispersoides ocupacionais para os homens. Para a faixa etária de 50 a 69 anos, sobressaem os agentes carcinogênicos ocupacionais, com aumento de 26.0% para homens e 17.1% para mulheres em 2016. Comparando o ranqueamento de 1990 e 2016 dos fatores de risco avaliados, os ocupacionais ascenderam de posição, com destaque em relação aos demais.

**CONCLUSÕES:**

A carga global de doenças crônicas não transmissíveis atribuídas aos fatores de risco ocupacionais têm adquirido importância crescente. Sugere-se reforçar a abordagem dos fatores de risco ocupacionais nas agendas para enfrentamento dessas doenças no Brasil.

## INTRODUÇÃO

As doenças crônicas não transmissíveis (DCNT) são causa de 41 milhões de mortes mundialmente. A cada ano, 15 milhões de pessoas na faixa etária de 30 a 69 anos morrem por doenças cardiovasculares, neoplasias, doenças respiratórias crônicas e diabetes. Alimentação inadequada, inatividade física, consumo abusivo de bebidas alcoólicas e tabagismo são comportamentos que aumentam o risco de DCNT. Esses riscos passíveis de modificação são alvo, no Brasil, do Plano de Ações Estratégicas para o Enfrentamento das DCNT^a^ , que está em sintonia com as diretrizes da Organização Mundial da Saúde (OMS)^1^ .

As DCNT são incapacitantes. No âmbito da Previdência Social (PS) brasileira, em 2016, as doenças osteomusculares foram a principal causa de afastamentos do trabalho, que justificaram 407 mil auxílios-doença, seguidas das doenças do aparelho digestivo (279 mil), doenças mentais (185 mil), neoplasias (165) e doenças do aparelho circulatório (159 mil)^b^ .

Há evidências de associação entre essas doenças e os fatores de risco ocupacionais^2 , 3 , 4 , 5^ . Em geral, são estudos transversais conduzidos em amostras de trabalhadores de um ramo ou setor da produção (indústria de material plástico^2^ , por exemplo) ou de categorias ocupacionais específicas como motoristas de ônibus^3^ ou profissionais da saúde^5^ . Esses e outros estudos foram comentados em recente artigo de revisão, em que os autores sistematizaram resultados dos inquéritos ocupacionais realizados na última década para obter um panorama tanto do aporte quanto das lacunas no conhecimento sobre a saúde dos trabalhadores brasileiros, além de indicar desafios no âmbito da saúde pública^6^ . Entre as lacunas e desafios, estão as dificuldades de estimar os riscos, em função da múltipla exposição nos ambientes laborais, bem como o subregistro relacionado à atual configuração do mercado de trabalho. A complexidade relacionada à múltipla exposição, em que ocorre combinação e, por vezes, sinergia dos efeitos, requer desenhos e métodos estatísticos sofisticados quando o objetivo é identificar os riscos e monitorar as situações laborais. Por essa e outras razões, a carga resultante dos riscos ocupacionais ainda não está suficientemente dimensionada, tampouco caracterizada de forma abrangente^7^ . Quanto ao subregistro, em sua origem está a incompletude ou ausência de informações sobre perfil de saúde, características de emprego e exposição ocupacional. As falhas de cobertura do sistema de informações sobre emprego são acentuadas diante da crescente proporção de pessoas ocupadas sem carteira assinada: 82,9% a 50,7%, como se observou no Maranhão e em Santa Catarina, respectivamente^c^ . Sabendo que 24,2% dos adultos brasileiros apresentam mais de uma doença crônica^8^ , como obter informações de qualidade para formular ações para monitorar e prevenir as DCNT associadas aos fatores de risco ocupacionais?

Avaliações exaustivas da saúde humana, desde os anos 1990, foram produzidas pelo estudo GBD, referente à sigla para o termo em inglês *Global Burden of Disease* . A principal métrica utilizada no estudo GBD é o *disability-adjusted life year* (DALY). Esse indicador, em uma única medida, expressa os anos saudáveis perdidos, ou seja, os anos de vida perdidos por morte prematura, e os anos não saudáveis, pois vividos com incapacidade. O cálculo desse indicador baseia-se em uma escala que varia de zero a um, em que zero designa o estado de plena saúde e um a morte, o pior estado de saúde possível. Os anos de vida perdidos por morte são calculados tomando como referência a esperança de vida estimada a partir das menores taxas de mortalidade existentes para cada grupo etário em locais com mais de cinco milhões de habitantes. Adicionam-se os anos perdidos por morte prematura ( *years of life lost* – YLL) aos anos vividos com incapacidade ( *years lived with disability* – YLD) por meio de uma escala que associa pesos relativos à mortalidade, às doenças e suas sequelas^9^ .

Além de medir perda de saúde, o estudo GBD examina três grupos de fatores de risco (FR). Para cada um daqueles que foram selecionados, calcula-se o risco atribuível populacional (RAP), que mede a carga de doença atribuível a determinadas exposições^10^ . O RAP permite identificar, por exemplo, quanto do risco total para câncer de pulmão fatal na população geral é devido aos riscos ocupacionais.

O objetivo do presente artigo foi utilizar as estimativas do estudo GBD 2016 para avaliar anos saudáveis de vida perdidos por morte prematura e incapacidade por DCNT atribuíveis a fatores de risco ocupacionais, além de comparar a posição desses fatores no ranqueamento dos riscos em 1990 e 2016.

## MATERIAL E MÉTODOS

### Contexto da Pesquisa

O avanço conceitual, metodológico e operacional promovido pelo estudo GBD resulta do esforço institucional de 195 países e territórios, além de contar com recursos computacionais potentes ( *big data* ) e financiamento garantido pela Fundação Bill & Melinda Gates^9^ . Para identificar tanto os riscos quanto a degradação da saúde por doenças e lesões, o estudo elabora estratégias complexas de combinação de dados de diferentes fontes. Os riscos selecionados são avaliados de forma articulada, de maneira a identificá-los de acordo com idade, sexo e área geográfica.

Desde a realização da oficina Carga de Doença em 2014 no Brasil, profissionais e pesquisadores da saúde pública estão implicados na parceria entre o *Institute for Health Metrics and Evaluation* (IHME)^10^ da Universidade de Washington (EUA), o Ministério da Saúde do Brasil e a Universidade Federal de Minas Gerais (UFMG). A utilização das métricas elaboradas pelo estudo GBD foi um dos impulsos para as instituições brasileiras aderirem à rede homônima^, 11^ . A difusão da metodologia alcançou o meio acadêmico e os serviços de saúde, de maneira a motivar, entre outros, a elaboração do presente artigo.

### Estimativas do Estudo GBD

As estimativas do estudo GBD resultam de um processo complexo de modelagem das fontes primárias, conforme suficientemente detalhado em anexo de publicação anterior^12^ . Softwares específicos foram desenvolvidos para estimar mortalidade (CODEM) e morbidade (Dis-mod), além de dispositivos técnicos para ajustes por covariáveis (PIB, escolaridade, etc.). As estimativas estão demonstradas com seus respectivos intervalos de incerteza. No site do IHME^10^ , o usuário acessa todas essas estimativas já calculadas por meio de metodologia padronizada. De acordo com o interesse do pesquisador, é possível tratar os dados já processados para 195 países, 20 grupos etários, ambos os sexos e três grupos de riscos: ambientais/ocupacionais, metabólicos e comportamentais. O usuário tem a opção de escolher a visualização de esquemas e figuras ilustrativas das informações de acordo com o ano de observação, risco e grupo de causa.

Conforme esclarecido anteriormente, interessa ao estudo GBD o risco atribuível populacional (RAP), que calcula o que seria reduzido na carga de doença se, no passado, a exposição tivesse sido modificada para um nível mínimo, o *theoretical minimum risk exposure level* (TMREL). O TRMEL é considerado um construto avançado, pois utiliza a exposição mínima com referência ao menor nível teórico de exposição. Além do TMREL, o RAP se baseia em outros dois componentes: o risco relativo e as prevalências dos fatores de risco na população. As estimativas de risco relativo são embasadas em resultados de pesquisas consistentes, como ensaios randomizados e controlados, estudos de coorte e outros, desde que desenvolvidos com métodos adequados^9^ . Os níveis de exposição e o risco relativo para cada um dos fatores elencados são medidos segundo a literatura disponível^12^ . As prevalências dos FR são estimadas de acordo com as informações originadas de diferentes tipos de pesquisas, como aquelas realizadas no domicílio do entrevistado ou medições ambientais coletadas por diferentes estratégias, incluindo satélite.

As definições do estudo GBD para cada FR ocupacional estão descritas no Quadro 1 .

**Quadro 1:** Definições dos fatores de risco ocupacionais avaliados pelo estudo GBD 13

Asbesto ocupacional	Exposição ocupacional acumulada ao asbesto, de acordo com a taxa de mortalidade por mesotelioma de pleura.
Substâncias broncoconstritoras no ambiente ocupacional	Proporção de indivíduos expostos a broncoconstritores no ambiente ocupacional, tendo como referência a distribuição da população em nove setores econômicos.
Carcinógenos ocupacionais	Proporção de indivíduos inseridos em grupos identificados como expostos (alta e baixa exposição) aos agentes carcinogênicos reconhecidos (arsênico, ácidos, benzeno, berílio, cádmio, cromo, diesel, formaldeído, níquel, hidrocarbonetos aromáticos policíclicos, tabagismo passivo, sílica, tricloroetileno), tendo como referência a distribuição da população em dezessete setores econômicos.
Acidentes de trabalho	Proporção de acidentes fatais atribuídos à atividade de trabalho em dezessete setores econômicos, tendo como referências taxas registradas em cada setor.
Fatores ergonômicos	Proporção de indivíduos expostos aos fatores de risco relacionados à lombalgia, tendo como referência a distribuição da população em nove setores econômicos.
Ruído ocupacional	Proporção de indivíduos expostos no ambiente ocupacional a nível de intensidade sonora superior a 85 decibéis, tendo como referência a distribuição da população em dezessete setores econômicos.
Partículas (aerodispersoides) no ambiente ocupacional	Proporção de indivíduos expostos a partículas dispersas no meio ambiente ocupacional, tendo como referência a distribuição da população em dezessete setores econômicos.

### Análise Apresentada neste Artigo

Este estudo descritivo utilizou as estimativas de carga global de doença para o Brasil do estudo GBD 2016, cujos dados estão disponíveis ao público no site do IHME^10^ , onde todos indicadores encontram-se calculados e atualizados. O foco deste estudo foi observar o indicador DALY para DCNT, de acordo com os FR desagregados até o terceiro nível^12^ . As proporções de anos saudáveis perdidos para homens e mulheres foram examinadas separadamente para dois grupos etários: 15 a 49 anos e 50 a 69 anos. Essa estratégia viabilizou obter o ranqueamento dos FR que mais contribuíram para o indicador DALY relacionado às DCNT, além de permitir a comparação dos resultados de 2016 com aqueles de 1990.

Sobre as DCNT, o estudo GBD inclui as doenças cardiovasculares, neoplasias malignas, outras neoplasias, doenças respiratórias, diabetes mellitus, doenças musculoesqueléticas, doenças da pele, doenças digestivas, doenças mentais e do sistema nervoso, doenças de órgãos sensoriais, doenças geniturinárias, condições neurológicas, anomalias congênitas, condições bucais e disfunções endócrinas, do sangue e imunológicas^13^ . Vale mencionar que os dados do estudo GBD 2016 sobre morbidade para o Brasil foram extraídos de inquéritos nacionais, como a Pesquisa Nacional de Saúde (PNS), a Vigilância de Fatores de Risco e Proteção para Doenças Crônicas por Inquérito Telefônico (Vigitel) e a Pesquisa Nacional por Amostra de Domicílios (PNAD), entre outros, totalizando 118 fontes. Para as fontes de exposição ocupacional, foram consultados resultados das pesquisas nacionais sobre a força de trabalho, estudos demográficos, informações dos sistemas internacionais sobre exposição ocupacional a agentes carcinogênicos, dados sobre acidentes de trabalho disponíveis nas bases da Organização Internacional do Trabalho e informações sobre ruído obtidas em inquéritos específicos realizados nas indústrias^13^ .

O projeto GBD Brasil 2015 foi aprovado pelo Comitê de Ética em Pesquisa da Universidade Federal de Minas Gerais, CAAE 62803316.7.0000.5149.

## RESULTADOS

Em 2016, na faixa etária entre 15 e 49 anos, considerada produtiva, o consumo de álcool e o IMC elevado foram os dois principais fatores de risco para os DALY por DCNT. Para os homens, prevaleceu o consumo de álcool (13.28%), e para as mulheres, o IMC elevado (7.77%). Quanto aos riscos ocupacionais, sobressaíram, nesta ordem, os fatores ergonômicos, os agentes carcinogênicos e o ruído, para ambos os sexos ( Figura 1 ).

**Figura 1:** Principais fatores de riscos para doenças crônicas não transmissíveis segundo anos de vida ajustados por incapacidade ( *disability-adjusted life years* – DALY), de acordo com sexo e faixa etária. Brasil, 2016.

Entre os homens de 15 a 49 anos, os fatores ergonômicos (2,57% dos DALY) ascenderam da 15ª posição, em 1990, para a 9ª posição, em 2016, perfazendo um aumento de 25,47% nos DALY. Vale mencionar que somente o aumento referente à dieta rica em sódio (84,64%) e ao consumo de drogas (30,7%) foi maior do que o referente aos FR ergonômicos ( Figura 2A ). Entre as mulheres ( Figura 2B ), esses FR (2,11%) ascenderam da 14ª para a 8ª posição, correspondendo ao maior aumento (22,39%) depois da dieta rica em sódio (40,99%). Já os agentes carcinogênicos ocupacionais (1,23%) entre os homens ascenderam da 19ª para a 14ª posição, perfazendo aumento de 11,89% em relação a 1990 ( Figura 2A ). Entre as mulheres, o aumento foi de 9,54% no total de DALY, provocando a mudança da 20ª para a 16ª posição em 2016 ( Figura 2B ). O terceiro risco ocupacional no ranqueamento de 2016 foi o ruído, que ascendeu da 21ª para 20ª posição e da 24ª para 22ª entre homens ( Figura 2A ) e mulheres ( Figura 2B ), respectivamente, na faixa etária de 15 a 49 anos. Em relação a 1990, o aumento do total de DALY por DCNT atribuíveis ao ruído ocupacional foi maior para as mulheres (20,38%) que para os homens (11,9%).

**Figura 2A:** Fatores de risco que mais contribuíram para a morte prematura e perda de saúde por doenças crônicas não transmissíveis entre homens de 15 a 49 anos. Brasil, 1990 e 2016 DALY: *disability-adjusted life years* (anos de vida ajustados por incapacidade)

**Figura 2B:** Fatores de risco que mais contribuíram para a morte prematura e perda de saúde por doenças crônicas não transmissíveis entre mulheres de 15 a 49 anos. Brasil, 1990 e 2016 DALY: *disability-adjusted life years* (anos de vida ajustados por incapacidade)

Na faixa etária entre 50 e 69 anos, a pressão arterial e o IMC elevados são os principais riscos para os DALY por DCNT. Para os homens, é mais relevante a pressão arterial elevada (19,55%). Para as mulheres, é o IMC elevado (17,1%). Na referida faixa, destacam-se os seguintes riscos ocupacionais em ambos os sexos, nesta ordem: agentes carcinogênicos ocupacionais, fatores ergonômicos e ruído ( Figura 1 ).

Entre os homens, na faixa etária entre 50 e 69 anos, agentes carcinogênicos ocupacionais (2,97%) ascenderam da 16ª posição, em 1990, para a 13ª posição, em 2016 ( Figura 3A ), perfazendo um aumento de 26,04% nos DALY. Entre as mulheres, na referida faixa etária, esses riscos (1,95%) ascenderam da 19ª para 12ª posição ( Figura 3B ), perfazendo um aumento de 17,1%. Quanto aos fatores ergonômicos, entre os homens (1,18%), eles ascenderam da 22ª para a 18ª posição ( Figura 3A ), perfazendo um aumento de 30,7%. Entre as mulheres (1,06%), a mudança foi da 22ª para a 20ª posição ( Figura 3B ), perfazendo um aumento de 14.43%. O terceiro risco ocupacional é o ruído, que, em 2016, ascendeu da 23ª para a 21ª posição entre os homens, um aumento de 19,27% em relação a 1990 ( Figura 3A ). Entre as mulheres, na faixa etária entre 50 e 69 anos, a posição mudou de 25º (1990) para 23º lugar (2016), perfazendo aumento de 19,39% ( Figura 3B ).

**Figura 3A:** Fatores de risco que mais contribuíram para a morte prematura e perda de saúde por doenças crônicas não transmissíveis entre homens de 50 a 69 anos. Brasil, 1990 e 2016 DALY: *disability-adjusted life years* (anos de vida ajustados por incapacidade)

**Figura 3B:** Fatores de risco que mais contribuíram para a morte prematura e perda de saúde por doenças crônicas não transmissíveis entre mulheres de 50 a 69 anos. Brasil, 1990 e 2016 DALY: *disability-adjusted life years* (anos de vida ajustados por incapacidade)

Os aerodispersoides (gases, vapores e partículas no ambiente) configuraram FR relevante somente para os homens de 50 a 69 anos em 1990. Observou-se, entretanto, descenso de posição desse risco, da 21ª para a 23ª posição.

Em síntese, em 1990 e 2016, o principal FR ocupacional para DCNT foi o ergonômico, na faixa etária de 15 a 49 anos, tanto em homens quanto em mulheres. Porém, quando analisada a faixa etária de 50 a 69 anos, em 1990 e 2016, o principal fator de risco ocupacional para DCNT são os agentes carcinogênicos ocupacionais, em ambos os sexos.

## DISCUSSÃO

Utilizando estimativas do estudo GBD, observou-se aumento, em 2016, dos anos saudáveis de vida perdidos por DCNT atribuíveis aos riscos ocupacionais em relação ao ano de 1990. O destaque dos riscos ergonômicos, agentes carcinogênicos e ruído não foi surpreendente, se considerados os conhecimentos sobre a associação entre esses riscos ocupacionais e o adoecimento dos indivíduos^2 , 3 , 4 , 5^ . Além disso, há evidências em outros países sobre a relação entre esses riscos e a carga de doença^14 , 15 , 16^ . As diferenças observadas quando examinados os anos saudáveis perdidos de acordo com o sexo confirmam hipóteses sobre a divisão sexual do trabalho^17^ .

No Irã, fatores ergonômicos e ruído lideraram a lista dos riscos ocupacionais que mais contribuíram para o total de DALY em 2015^16^ . Na Espanha, os agentes carcinogênicos ocupacionais ocuparam a 12ª posição no ranqueamento dos riscos ambientais relacionados ao total de DALY em 2016^15^ . Na Índia, os riscos ocupacionais ocuparam a terceira posição quando calculados os anos saudáveis perdidos em função das doenças pulmonares obstrutivas crônicas em 2016^14^ . As disparidades verificadas nas comparações podem ser imputadas a um conjunto de fatores que varia de um país para outro – por exemplo, as diferenças quanto à qualidade dos sistemas de informação nacionais. No estudo iraniano, as estimativas não incluíram a exposição ao cancerígeno tricloroetileno, porque essa informação não estava disponível em fonte confiável no país^16^ . Quanto ao estudo realizado na Espanha, Soriano et al.^15^ apresentaram uma síntese da situação de saúde, de acordo com as estimativas do estudo GBD para o país em 2016 para todas as 333 enfermidades e lesões, distinguindo-se, portanto, do presente estudo, que se focou apenas nas DCNT. No estudo realizado na Índia, as estimativas dizem respeito exclusivamente à doença pulmonar obstrutiva crônica (DPOC) e à asma^14^ . Vale mencionar a necessária cautela na interpretação sobre as disparidades, uma vez conhecidos os limites específicos dos estudos sobre doenças respiratórias e outras DCNT, como ausência de consenso na definição de caso e multiplicidade de procedimentos na realização da espirometria, além do viés provocado por barreiras de acesso aos serviços onde os diagnósticos são realizados. No plano técnico, procedimentos complexos para o dimensionamento da população exposta são recomendados quando é longo o período de latência de uma doença, como é o caso do câncer. As capacidades nacionais variam também no tocante a tais insumos. Além disso, erros na avaliação da exposição podem influenciar os resultados de um estudo nacional^18^ . Esse conjunto de fatores explicariam, ao menos em parte, as diferenças nas estimativas. Mais adiante, serão apresentados os limites do estudo GBD, que convém evocar quando se analisa o contraste dos resultados interpaíses.

Fatores macroestruturais explicariam as diferenças no ranqueamento dos riscos ocupacionais. Sabe-se das particularidades nacionais quanto à rotatividade de emprego e expectativa de vida, que influenciam a idade na entrada e saída na força de trabalho, com repercussões sobre o tamanho e vulnerabilidade da população exposta aos carcinógenos ocupacionais, por exemplo. Apesar das disparidades quando comparados os resultados nacionais, as consistências quando examinados dados correlatos (as informações da PS sobre causas de afastamento do trabalho, por exemplo) são evidências indispensáveis para interpretar os resultados descritos. Conforme mencionado, em 2016, predominaram os auxílios-doença por causas musculoesqueléticas, as quais, na população adulta, são comumente associadas aos fatores ergonômicos^19^ . Tem-se assim uma coerência entre carga de doença atribuível a riscos ergonômicos, conforme indicam os resultados do presente estudo, e as doenças musculoesqueléticas como primeira causa de afastamentos laborais reconhecidos e declarados pela PS em 2016.

Recortes de gênero explicam a composição da força de trabalho, de acordo com o sexo, o que se denomina divisão sexual do trabalho^17^ . No Brasil, em 2014, na construção civil estavam inseridos 15,6% dos homens ocupados. Na indústria estavam 17,2% dos homens ocupados e 10,6% das mulheres ocupadas. No setor de serviços (educação, saúde e serviços sociais), a proporção foi inversa: nele encontravam-se 4,3% dos homens ocupados, contra 18,2% das mulheres nessa situação. Ainda em relação às mulheres ocupadas, a maioria realizava serviços domésticos (13,9%) ou se encontrava laborando no setor educacional (7,4%). Veja-se assim que, apesar de ser crescente a participação das mulheres na força de trabalho, elas continuam em minoria na indústria metalúrgica pesada, na mineração, na construção civil e no setor agrícola, para citar alguns^d^ . Essa divisão sexual do trabalho reproduz configurações de identidade masculina e feminina. Os homens predominam em setores nos quais as tarefas exigem mais força física e contato com substâncias químicas. As mulheres são maioria no setor de serviços caracterizados pela prestação de cuidados. Na indústria, elas ocupam postos caracterizados pelas operações mais minuciosas e repetitivas, como é o caso da microeletrônica^17^ . Com essa distribuição de postos e tarefas espera-se coincidir a distribuição da exposição aos riscos ocupacionais específicos de um e outro setor, de maneira a elucidar os diferenciais da carga de doença atribuível a esses riscos de acordo com o sexo^20^ . De forma similar, em outros países, o ranqueamento dos riscos ocupacionais mostrou-se afetado pela variável sexo^14 , 15 , 16^ .

Quanto aos agentes carcinogênicos, observou-se maior relevância no ranqueamento dos riscos para os homens que para as mulheres, principalmente na faixa etária mais jovem. Mais uma vez as hipóteses sobre divisão sexual do trabalho^20^ auxiliam na interpretação dos resultados, porque a maioria dos carcinogênicos presentes nos ambientes laborais são substâncias químicas (amianto, benzeno e organofosforados, por exemplo), manuseadas principalmente na metalúrgica pesada, mineração, construção civil e setor agrícola, setores majoritariamente masculinos^4^ . Na faixa etária de 50 a 69 anos, o destaque dos riscos carcinogênicos em relação aos demais riscos ocupacionais foi observado independentemente do sexo. O lento processo da carcinogênese desencadeado pela exposição ocupacional continuada a tais agentes^e^ explicaria o aumento progressivo das taxas de incidência e mortalidade, de acordo com a idade, para homens e mulheres.

O ruído é o terceiro FR ocupacional identificado. Esse resultado é coerente com a classificação da Organização Mundial de Saúde^f^ , que o posiciona em terceiro lugar entre os riscos ocupacionais. Sabe-se que ruído de alta intensidade e baixa frequência é produzido por motores, bombas, sistemas de ventilação etc., principalmente nos setores de transporte, indústria, mineração, construção civil e agrícola^21^ . Os efeitos das alterações das funções auditivas e extra-auditivas induzidas pelo ruído produzido nas instalações dos setores mencionados, além de outros, estão claramente associados à prevalência de DCNT entre os expostos^22^ .

O aumento da carga de doença atribuível aos riscos ocupacionais para as DCNT, em 2016, seria o efeito do período marcado por mudanças institucionais, sociais e econômicas que modificaram os ambientes laborais e os vínculos de emprego? No plano institucional, houve no Brasil substancial transformação das políticas sociais nas últimas décadas, levando ao avanço do conhecimento e da atenção à saúde, incluindo a saúde do trabalhador. Ampliação do acesso e melhoria da qualidade dos sistemas de informação são resultados dessas políticas, que beneficiam o diagnóstico dos eventos e o registro dos riscos^1^ . Mas é possível que o aumento da contribuição dos riscos ocupacionais na carga de doença seja, em sintonia com as hipóteses da presente pesquisa, uma decorrência da deterioração dos ambientes laborais com as inovações dos processos produtivos e precariedade das relações trabalho. No plano macroestrutural, o capitalismo globalizado, nestes tempos que correm, confronta cada vez mais o trabalho e os trabalhadores. Mudanças nos ambientes laborais com a introdução de novas tecnologias, substâncias e processos de trabalho são tão velozes quanto o *design* comercial e o incentivo para o consumo de novos produtos em escala mundial^23^ . Essa transformação está acompanhada da exposição a novos riscos e aumento da exposição àqueles já conhecidos^7^ .

Os dados do estudo GBD para o Brasil viabilizaram estimar, em caráter inédito, o impacto dos riscos ocupacionais sobre as DCNT em adultos brasileiros, por meio de métricas e construtos potentes e inovadores (DALY e RAP, por exemplo), de maneira a contornar alguns limites dos estudos clássicos^6^ . Ao reunir fontes de dados nacionais, principalmente os resultados dos inquéritos domiciliares, além de medições por satélite, o estudo GBD criou oportunidades para os pesquisadores e profissionais da saúde pública. Os limites conhecidos do estudo GBD, contudo, devem ser mencionados. As estimativas de risco talvez estejam provocando subestimação do efeito e, portanto, da carga^24^ . Em primeiro lugar, sabe-se que as informações nacionais sobre o número de indivíduos expostos e níveis de exposição são geralmente incompletas ou inexistentes, seja pela heterogeneidade da distribuição da força de trabalho, seja pela diversidade de arranjos dos setores e subsetores da produção, nos quais a exposição a múltiplos agentes torna bastante complexa a mensuração dos riscos^4 , 7 , 25^ , conforme esclarecido anteriormente. Em segundo lugar, ainda que o monitoramento de fatores de risco ocupacionais, mensurações ambientais e controle periódico dos trabalhadores potencialmente expostos estejam previstos no marco regulatório^g^ , não existem sistemas de informação para exposições ocupacionais no Brasil. A rotatividade no emprego e o efeito do trabalhador sadio constituem, em terceiro lugar, mais uma barreira, porque a tendência é a exclusão daqueles com menos saúde para enfrentar as demandas da produção. A rotatividade explica a pulverização da exposição, haja vista a mudança de empresa ou de setor^4 , 6 , 25^ . Além disso, está reconhecida internacionalmente a insuficiência do investimento intelectual, acadêmico e financeiro necessário para identificar a carga de doença relacionada aos problemas ocupacionais. Nesse cenário da dinâmica do emprego e escassez de informações, encontra-se prejudicada a elaboração de medidas de vigilância à saúde dos trabalhadores, com repercussões sobre as chances de adoecimento^7^ .

A relevância dos riscos ocupacionais na carga de doença, conforme evidenciam os resultados apresentados, está em sintonia com publicações anteriores no Brasil^26^ e em outros países^14 , 15 , 16^ . As doze metas nacionais do Plano de Ações Estratégicas para o Enfrentamento das Doenças Crônicas Não Transmissíveis (2011–2022), entretanto, não incluem a abordagem da exposição ocupacional. Ora, a Política Nacional de Saúde do Trabalhador e da Trabalhadora^h^ , a seu turno, explicita a intenção de alinhar-se ao conjunto de políticas de saúde no âmbito do Sistema Único de Saúde, uma vez admitido o trabalho como um dos determinantes do processo saúde-doença^7 , 27^ . Os resultados inéditos sobre o impacto das DCNT atribuíveis aos riscos ocupacionais provavelmente suscitarão debates sobre a almejada transversalidade das ações de saúde do trabalhador, além de fornecer elementos para a elaboração de eixos relacionados à ocupação nas próximas atualizações das políticas para monitorar e prevenir as DCNT.

## CONCLUSÃO

Analisando os três grupos de fatores de risco para DALY por DCNT em1990 e 2016, observou-se mudança da posição dos riscos ocupacionais em relação aos demais FR avaliados no estudo GBD, em ambos os sexos. O destaque conferido pelos FR ocupacionais provavelmente está relacionado às mudanças estruturais no mercado de trabalho e nos processos produtivos. A divisão sexual do trabalho explicou as diferenças no ranqueamento dos FR de acordo com o sexo. Os resultados apresentados estão em sintonia com o conhecimento sobre as associações entre DCNT e exposição ocupacional, além de convergentes com as causas de incapacidade, no mesmo período, de adultos cobertos pela seguridade social. A elaboração de eixos relacionados à ocupação nas próximas atualizações das políticas para monitorar e prevenir as DCNT é desejável.

## Footnotes

a Brasil. Ministério da Saúde. Secretaria de Vigilância em Saúde. Departamento de Análise de Situação de Saúde. Plano de ações estratégicas para o enfrentamento das doenças crônicas não transmissíveis (DCNT) no Brasil 2011-2022 / Ministério da Saúde. Secretaria de Vigilância em Saúde. Departamento de Análise de Situação de Saúde. – Brasília: Ministério da Saúde, 2011. Acesso 28 Ago 2018.

b Ministério da Fazenda. Anuário Estatístico da Previdência Social/Ministério da Fazenda. Secretaria de Previdência, Empresa de Tecnologia e Informações da Previdência. Brasília: MF/DATAPREV, 2016. Acesso 22 Fev 2019.

c Instituto Brasileiro de Geografia e Estatística. Síntese de indicadores sociais: uma análise das condições de vida da população brasileira: 2016 / IBGE, Coordenação de População e Indicadores Sociais. - Rio de Janeiro: IBGE, 2016. Acesso 24 Fev 2019.

d Departamento Intersindical de Estatística e Estudos Socioeconômicos (DIEESE). Anuário do Sistema Público de Emprego, Trabalho e Renda 2015: mercado de trabalho. São Paulo: DIEESE, 2015.

e Brasil. Ministério da Saúde. Secretaria de Vigilância em Saúde. Departamento de Vigilância Ambiental e Saúde do Trabalhador. Atlas do câncer relacionado ao trabalho no Brasil. Brasília. Ministério da Saúde, 2019. Disponível em: http://bvsms.saude.gov.br/bvs/publicacoes/atlas_cancer_relacionado_trabalho_brasil.pdf . Acesso 24 Fev 2019.

f WHO - World Health Organization. Global Health Risks: Mortality and burden of disease attributable to selected major risks. WHO: Geneva, 2009. Acesso 12 Set 2018.

g Brasil. Ministério da Saúde. Secretaria de Vigilância em Saúde. Departamento de Vigilância Ambiental e Saúde do Trabalhador. Atlas do câncer relacionado ao trabalho no Brasil. Brasília. Ministério da Saúde, 2019. Disponível em: http://bvsms.saude.gov.br/bvs/publicacoes/atlas_cancer_relacionado_trabalho_brasil.pdf . Acesso 24 Fev 2019.

h Brasil. Ministério da Saúde. Portaria nº 1.823, de 23 de agosto de 2012: Institui a Política Nacional de Saúde do Trabalhador e da Trabalhadora. Disponível em: http://bvsms.saude.gov.br/bvs/saudelegis/gm/2012/prt1823_23_08_2012.html . Acesso em 24 Fev 2019.
